# Structural Insights into the *Giardia lamblia* Target of Rapamycin Homolog: A Bioinformatics Approach

**DOI:** 10.3390/ijms241511992

**Published:** 2023-07-26

**Authors:** Patricia L. A. Muñoz-Muñoz, Rosa E. Mares-Alejandre, Samuel G. Meléndez-López, Marco A. Ramos-Ibarra

**Affiliations:** Biotechnology and Biosciences Research Group, School of Chemical Sciences and Engineering, Autonomous University of Baja California, Tijuana 22390, Mexico; lilian.munoz.munoz@uabc.edu.mx (P.L.A.M.-M.); samuelmelendez@uabc.edu.mx (S.G.M.-L.); mramos@uabc.edu.mx (M.A.R.-I.)

**Keywords:** template-based protein modeling, structure–function computational analysis, target of rapamycin, *Giardia lamblia*

## Abstract

TOR proteins, also known as targets of rapamycin, are serine/threonine kinases involved in various signaling pathways that regulate cell growth. The protozoan parasite *Giardia lamblia* is the causative agent of giardiasis, a neglected infectious disease in humans. In this study, we used a bioinformatics approach to examine the structural features of GTOR, a *G. lamblia* TOR-like protein, and predict functional associations. Our findings confirmed that it shares significant similarities with functional TOR kinases, including a binding domain for the FKBP-rapamycin complex and a kinase domain resembling that of phosphatidylinositol 3-kinase-related kinases. In addition, it can form multiprotein complexes such as TORC1 and TORC2. These results provide valuable insights into the structure–function relationship of GTOR, highlighting its potential as a molecular target for controlling *G. lamblia* cell proliferation. Furthermore, our study represents a step toward rational drug design for specific anti-giardiasis therapeutic agents.

## 1. Introduction

TOR proteins, also known as targets of rapamycin, are a class of Ser/Thr kinases that play critical roles in regulating cell growth by integrating environmental and nutritional signals. This kinase family is conserved from yeast to humans and comprises proteins with a canonical domain organization: HEAT–FAT–FRB–PIKKc–FATC [1,2,3]. Rapamycin, a natural antifungal antibiotic, binds to FKBP (FK506-binding protein) and interacts with TOR proteins via the FKBP–rapamycin binding (FRB) domain [4,5]. Notably, this binary complex exhibits a higher affinity for the FRB domain than rapamycin alone, indicating that FKBP is crucial for rapamycin binding and that protein interactions are essential for stabilizing the FKBP–rapamycin–TOR complex [6]. Therefore, TOR cannot form functional multiprotein complexes such as TORC1 and TORC2 [6,7].

*Giardia lamblia* is the protozoan parasite that causes human giardiasis, an intestinal infection that can lead to severe diarrhea. It ranks among the top ten human parasites worldwide [8,9,10]. Giardiasis is a disease with a global distribution. However, it is more prevalent in low-income countries with poor hygiene practices [11]. Moreover, because poverty and disease burden are associated, it has been included in the WHO Neglected Diseases Initiative [12].

Metronidazole and other nitroimidazole derivatives are usually prescribed to treat human giardiasis [13,14]. However, poor patient compliance with drug therapy and rapid reinfection in endemic areas have raised concerns regarding the emergence of resistant strains. Additionally, limited therapeutic options have intensified this situation. Thus, it is imperative to discover novel anti-giardiasis agents that can improve patient outcomes and reduce the likelihood of drug resistance [13,15,16].

A critical initial step toward developing new or improved drugs to treat infectious diseases is identifying reliable molecular targets, such as unique virulence factors or well-known proteins involved in essential processes [17,18,19]. TOR kinases are valuable targets because of their functional conservation and implications in cell biology [2]. In this study, we investigated the *G. lamblia* TOR protein (GTOR) using a biocomputational approach to gain further insights into its structural features and potential for inhibition. Our research findings demonstrated that it has domains with a highly similar structure to that of functional TOR kinases. Consequently, it seems feasible to presume that GTOR represents a viable target for developing specific therapeutic agents using a rational approach such as structure-based drug design.

## 2. Results and Discussion

### 2.1. GTOR, a TOR-like Protein Encoded by G. lamblia

**Figure 1:**
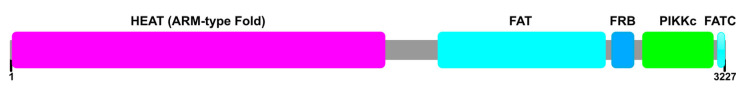
Graphic representation of the GTOR domain organization. HEAT/ARM (8–1689; magenta), FAT (1931–2684; cyan), FRB (2717–2815; blue), PIKKc (2859–3172; green), and FATC (3195–3227; cyan). Illustration generated using the IBS program [21].

A bioinformatic approach was used to investigate the structural features of GTOR, a 363 kDa TOR-like protein encoded by the human parasite *G. lamblia*. Primary structure analysis revealed that GTOR exhibits a domain organization similar to that of active TOR kinases (Figure 1): an amino-terminal region comprising HEAT repeats with a predicted armadillo (ARM)-type fold and a carboxy-terminal region that contains the FRB–PIKKc domain segment wedged between the FAT and FATC domains. This domain arrangement suggests proper kinase function [20].

### 2.2. The FRB Domain of GTOR Has Relevant Features

**Figure 2:**
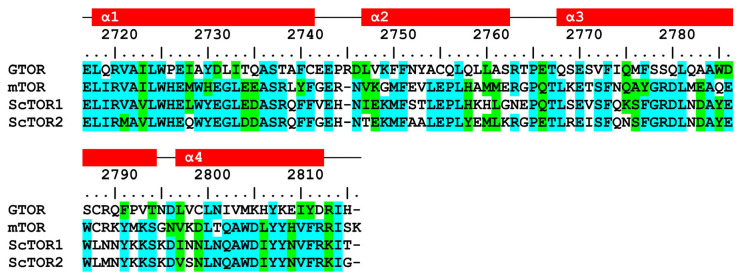
Multiple-sequence alignment of FRB domains: GTOR (2717–2815), mTOR (2015–2113), ScTOR1 (1952–2049), and ScTOR2 (1955–2052). Residues shaded in cyan are identical, whereas those shaded in green are similar. At the top, the residue numbering corresponds to the GTOR protein, and the rectangles represent the predicted 2D structure (red, α-helix).

A multiple-sequence alignment generated initial data on the conserved functional residues in the FRB domain of GTOR. As suspected, this domain is 28–29% identical to its human and yeast counterparts, showing a typical 2D structure consisting of four α-helices (Figure 2). Moreover, compared with human mTOR, it retains residues that putatively interact with the FKBP–rapamycin complex [5,22]: A2729, I2733, S2737, F2740, C2741, N2801, M2804, K2805, K2808, and Y2811.

**Figure 3:**
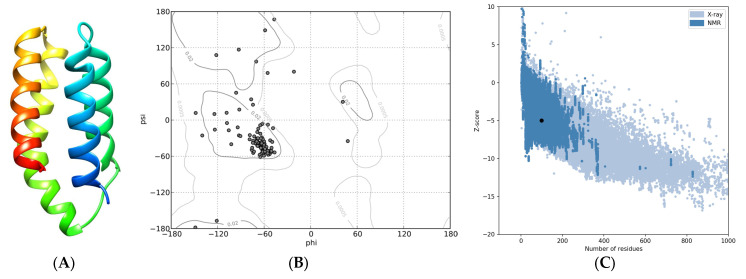
Tertiary structure of the FRB domain included in GTOR. (**A**) Ribbon representation of the best 3D model, rainbow-colored (blue to red) from the N to the C terminus. (**B**) Ramachandran plot. (**C**) ProSA plot: The black dot indicates the estimated Z-score.

Homology-based modeling of the FRB domain provided additional information on the structure–function relationship. The best model (Figure 3A), which displayed a 3D structure with ≥97% residues in the most favored regions (Figure 3B), a Z-score of −5.0 (Figure 3C), and a MolProbity Clashscore of 4.34 (96th percentile), confirmed the four-helix bundle pattern with the amino and carboxy termini close to each other.

**Figure 4:**
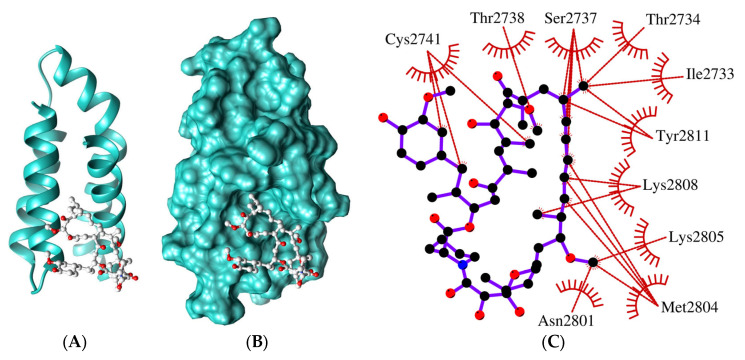
Ligand-binding site of the FRB domain. Best 3D model representations: ribbon (**A**) and surface (**B**). Rapamycin (the ligand), represented by balls and sticks, is colored according to the elements. (**C**) Two-dimensional illustration of the putative rapamycin-interacting residues. Colors: hydrophobic interactions, red dashes/arcs; carbon, black; oxygen, red; nitrogen, blue; ligand bonds, purple.

Further analyses revealed that two helices, α1 and α4, constitute a ligand-binding cleft that includes various residues that can interact with rapamycin (Figure 4), resembling the ligand-binding site of mTOR [23]. This observation suggested that well-known ligands can block the FRB domain of GTOR. Moreover, it highlights the prospect of regulating TOR function with specific molecules targeting the FRB domain without FKBP [24], which can be explored to develop new or improved anti-giardiasis therapeutic drugs.

### 2.3. GTOR Contains a Conserved PIKKc Domain

**Figure 5:**
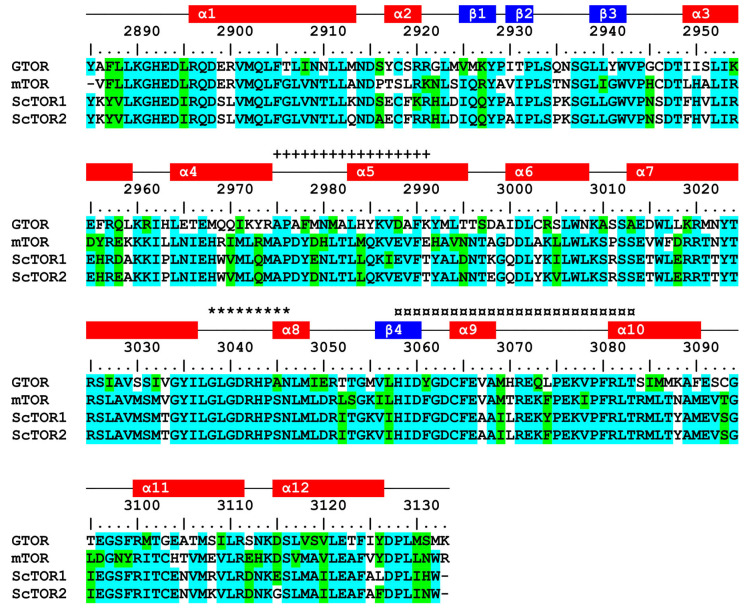
Multiple-sequence alignment of PIKKc domains. GTOR (2895–3133), mTOR (2183–2430), ScTOR1 (2119–2366), and ScTOR2 (2123–2370). Residue shading and numbering are shown in Figure 2. The colored rectangles represent the predicted secondary structures (red: α-helix, blue: β-sheet). Additionally, the LST8 interface (+), catalytic loop (*), and activation loop (¤) are indicated.

Multiple-sequence alignment showed that GTOR has a conserved PIKKc domain that is significantly identical (52–54%) to its human and yeast counterparts. Further primary and secondary structural analyses revealed two known motifs, catalytic and activation loops, and the expected LST8 interface (Figure 5). Moreover, it includes three residues that are critical for kinase function: D3041, which plays a crucial role in substrate orientation and activation for nucleophilic attack; H3043, which participates in stabilizing the buildup of the charge in the transition state; and N3046, which serves as a metal-ligand [23].

**Figure 6:**
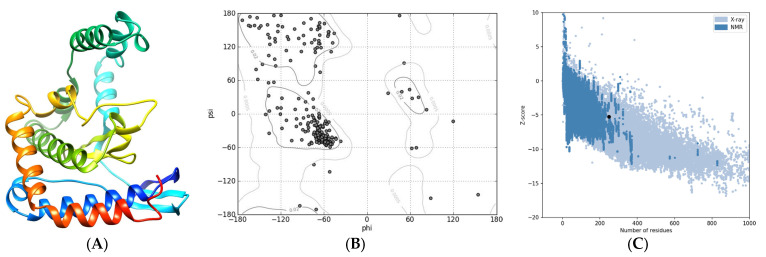
Tertiary structure of the PIKKc domain included in GTOR. (**A**) Ribbon representation of the best model, rainbow-colored (blue to red) from the N to the C terminus. (**B**) Ramachandran plot. (**C**) ProSA plot: The black dot indicates the estimated Z-score.

**Figure 7:**
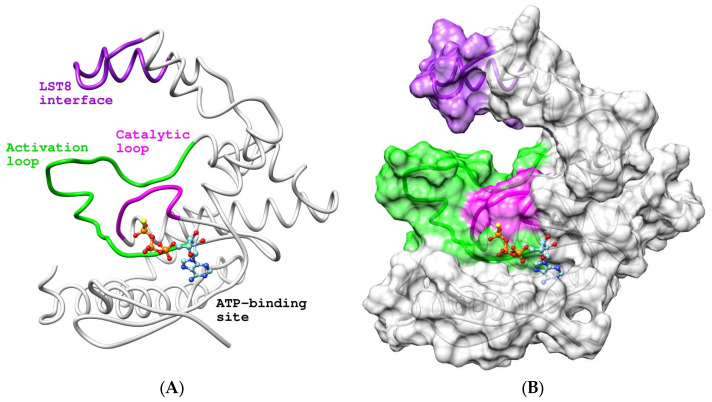
Features of the GTOR PIKKc domain. Licorice (**A**) and surface (**B**) representations of the best model showing the ATP-binding site, kinase-associated loops: activation (green) and catalytic (magenta), and the LST8 interface (purple). The theoretical pose of a non-hydrolyzable ATP analog (ATPγS), displayed as element-colored balls and sticks, was predicted via comparative 3D structure pairwise alignment using the 4JSP crystal as a template.

Homology-based modeling provided additional insights into the structure–function relationship of the PIKKc domain. The best model (Figure 6A) confirmed a kinase folding pattern, showing a 3D structure with ≥97% residues in the most favored regions (Figure 6B), a Z-score of −5.3 (Figure 6C), and a MolProbity Clashscore of 2.23 (99th percentile). A comparative analysis of 3D structures, conducted through pairwise alignment and employing the mTORΔN–mLST8–ATPγS–Mg complex as a template (PDB: 4JSP [23]; RMSD of 0.6 Å), showed that the ATP-binding site is near the catalytic and activation loops (Figure 7), with several conserved residues potentially interacting with ATP: L2888, K2890, E2893, V2943, N3046, M3048, and D3060. Additionally, the location of the LST8 interface was consistent with the predicted structural motif.

Inhibition of the kinase activity of TOR proteins is a feasible approach for blocking the functions of both TORC1 and TORC2 complexes, as evidenced by promising results from studies on treating certain types of cancers with ATP-competitive inhibitors of mTOR kinase activity [25,26,27,28,29]. Therefore, it seems reasonable to postulate that the ATP-binding site of the PIKKc domain is a reliable drug target for developing GTOR-specific kinase inhibitors, which may represent effective therapeutic agents to control giardiasis by disrupting the TOR signaling pathway, thus impairing *G. lamblia* metabolism and cell proliferation.

### 2.4. GTOR Participates in PPI Networks

**Figure 8:**
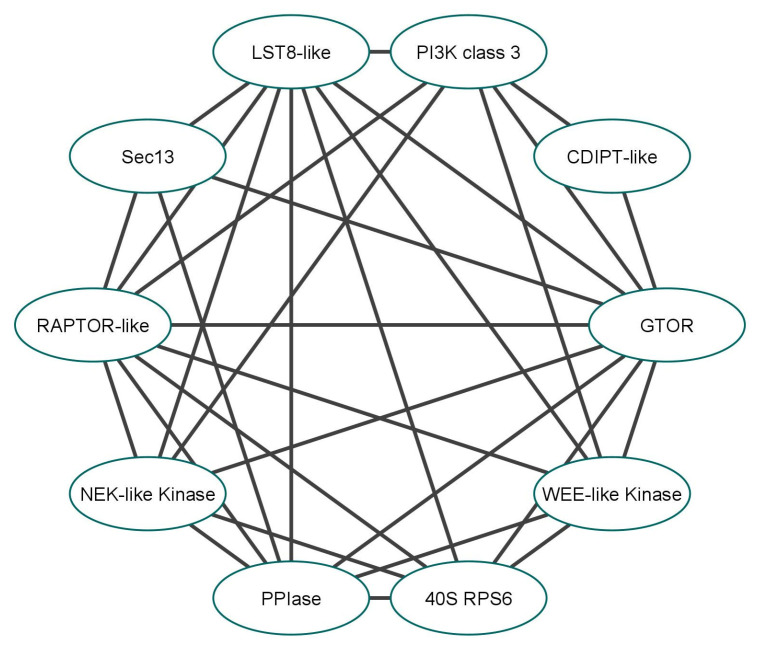
PPI network predicted for GTOR. Interaction partners (UniProtKB entry code): WEE-like kinase (A8B810); 40S ribosomal protein S6, 40S RPS6 (V6TX01); peptidyl-prolyl isomerase, PPIase (V6TV88); NEK-like kinase (A8BNA4, A8BPN1); regulatory-associated protein of TOR (RAPTOR)-like (A8B621); Sec13 (A8B9T4); lethal with Sec13 protein 8 (LST8)-like (V6TT55); phosphoinositide-3-kinase (PI3K) class 3 (A8B8M9); cytidine diphosphate-diacylglycerol-inositol-3-phosphatidyltransferase (CDIPT)-like (A8BGD6).

Analysis of the predicted protein–protein interaction (PPI) network provided further data regarding the ability of GTOR to bind or interact with putative TORC components or other *G. lamblia* proteins involved in TOR-linked signaling pathways (Figure 8).

GTOR can potentially interact with various proteins, including two putative TORC components, LST8- and RAPTOR-like proteins, and a PPIase (known to bind rapamycin [30,31]), which share significant similarities with their corresponding yeast and human orthologs: 44–49% for LST8, 40–44% for RAPTOR, and 61–67% for PPIase. Furthermore, other proteins, such as NEK and WEE kinases, PI3K, CDP-DAG-inositol-3-phosphatidyltransferase, 40S ribosomal protein S6, and Sec13, are presumed to be GTOR partners. However, additional studies are required to establish their precise functions in the TORC-related pathways in *G. lamblia*.

### 2.5. TORC1 and TORC2 in G. lamblia: In Silico Identification

Supplementary biocomputational analyses provided further information regarding TOR complexes in *G. lamblia*, denoted as GTORC1 and GTORC2. Both complexes must contain GTOR (the only TOR-like protein encoded by this human protozoan). Moreover, as observed in their mammalian counterparts [32], they contain a minimal protein core for proper substrate-specific recognition. GTORC1 includes the GTOR/RAPTOR/LST8 ensemble, whereas GTORC2 involves the GTOR/RICTOR/LST8 cluster (Table 1). Furthermore, a RICTOR-like protein (undetected by BLAST and STRING analyses) was identified through an extensive search using UniProtKB (D3KGC1). In contrast, the absence of other GTORC2-specific components (such as the Avo1/mSIN1 homolog) suggests that protein identification using typical bioinformatic tools is not always the best approach. Therefore, biochemical isolation and analysis of both complexes are required to gain further insight into their structural composition and functional roles in the pathobiology of *G. lamblia*.

### 2.6. Final Remark: Is GTOR a Promising Drug Target?

Several studies have provided insights into the structural and functional regulation of TOR signaling, its multiprotein complexes, and the crosstalk with other signaling pathways [33,34,35,36,37]. In addition, TOR inhibitors have been developed as potential drugs for various diseases, including cancer, and the elucidation of TOR biology continues to be an active area of research in biology and medicine [38,39,40]. A recent study showed that the treatment of *G. lamblia* cells with 36 μM rapamycin reduced encystation, the process by which the parasite evolves from the replicative form (trophozoite) to the dormant stage (cyst) and induces cell death at higher concentrations (EC_50_ of 65–70 μM) [41]. Although rapamycin was used as a putative regulator of autophagy, these findings suggest that the TOR pathway is essential for cell growth and its disruption can block the parasitic life cycle. Therefore, it is reasonable to propose that targeting GTOR is a promising approach for developing new or improved drugs against human giardiasis, a neglected infectious disease.

## 3. Materials and Methods

### 3.1. Primary and Secondary Structure Analysis

The GTOR polypeptide sequence (entry code A8BIV9) was obtained from UniProtKB (https://www.uniprot.org/; accessed on 1 July 2022) [42]. The physicochemical parameters were estimated utilizing the ProtParam tool (https://web.expasy.org/protparam/; accessed on 1 July 2022) [43], while the sequence patterns associated with protein domains and families were detected using the ScanProsite tool (https://prosite.expasy.org/scanprosite/; accessed on 1 July 2022) [44,45,46]. The HEAT repeats were identified using the REP2 server (http://cbdm-01.zdv.uni-mainz.de/~munoz/rep/; accessed on 2 July 2022) [47], while the conserved and potentially functional domains were detected using the CD-Search engine (https://www.ncbi.nlm.nih.gov/Structure/cdd/cdd.shtml; accessed on 1 July 2022) [48,49]. The polypeptide architecture and organization were examined utilizing the Pfam tools (https://pfam.xfam.org/; accessed on 2 July 2022) [50], and the multi-sequence alignments were generated using the ClustalO program (https://www.ebi.ac.uk/Tools/msa/clustalo/; accessed on 5 July 2022) [51]. The secondary (2D) structure was predicted using the Ali2D tool (https://toolkit.tuebingen.mpg.de/; accessed on 5 December 2022) [52].

### 3.2. General Approach for the Modeling and Validation of 3D Protein Structures

First, a comparative analysis of the 3D models generated using different resources for protein structure prediction, I-TASSER [53,54], IntFOLD [55], Modeller [56,57], and AlphaFold2 [58,59], was performed. At this stage, the main criteria for selecting the best model were the Ramachandran plot and MolProbity outputs [60]. The Ramachandran plot is one of the most useful tools for validating protein structures, showing the mapping of pairs of φ/ψ torsion angles of the polypeptide backbone [60,61]. MolProbity is a widely used general-purpose system for validating the quality of protein models, that produces several outputs, including summary statistics of all-atom contacts and geometry [60,62]. Next, the protein structure was refined using two well-established tools: ModRefiner (an atomic-level algorithm for high-resolution refinement [63]) and FG-MD (an algorithm based on molecular dynamics for atomic-level refinement [64]). Finally, the structural quality was assessed using at least three algorithms: MolProbity, PROCHECK [65], Verify3D [66], and ERRAT [67], along with the Ramachandran plot and ProSA analysis [68].

### 3.3. Homology-Based Modeling of the FRB and PIKKc Domains

The 3D structure of two conserved and potentially functional GTOR domains (FRB and PIKKc) was predicted using I-TASSER (https://zhanggroup.org/I-TASSER/; accessed on 10 December 2022), one of the most widely used servers for automatic homology-based modeling. The top-ranked 3D structures were further improved using ModRefiner and FG-MD on the I-TASSER server. The accuracy of the best 3D structures was validated utilizing MolProbity (http://molprobity.manchester.ac.uk/; 12 December 2022), which combined the all-atom contact analysis with updated versions of more traditional tools to validate geometry and dihedral angle combinations [69,70]. The Ramachandran plot and ProSA (https://prosa.services.came.sbg.ac.at/; accessed on 12 December) were used for further validation. Unless otherwise stated, 3D structures were analyzed using the UCSF Chimera as a molecular visualization system [71].

### 3.4. Bioinformatic Analysis of the Rapamycin Binding Site

The putative rapamycin binding site was detected by primary and tertiary structure analysis of the FRB domain (2717–2815 residues) utilizing three bioinformatics tools. The IntFOLD suite (https://www.reading.ac.uk/bioinf/IntFOLD/; accessed on 9 January 2022), which predicts the binding site of target proteins through comparisons with ligand-containing PDB templates [55,72], was used to identify residues in FRB with the potential to bind rapamycin and their interactions (i.e., a 3D model of the most likely protein–ligand pose). This outcome was validated using COACH (https://zhanggroup.org/COACH/; accessed on 15 January 2022), a consensus approach to ligand binding site prediction that combines the results of five individual algorithms via the support vector machine (SVM) training [73,74], and PrankWeb (https://prankweb.cz/; accessed on 24 January 2022), a machine learning-based method for the prediction of ligand binding sites from protein structures [75]. The protein–ligand complex (3D structure) was visualized using UCSF Chimera and MolStar Viewer [76], whereas rapamycin-interacting residues were analyzed using LigPlot [77,78] and PLIP [79].

### 3.5. In Silico Prediction of Protein–Protein Interactions

The potential PPI partners of GTOR were detected using the STRING web resources (https://string-db.org/; accessed on 12 February 2023). The STRING database collects, scores, and integrates all available data on known and predicted PPIs [80,81]. The most probable PPI network was assembled using Cytoscape 3.9.1 [82], with 0.7 as a benchmark for high confidence. All predicted interacting partners were further analyzed using the InterPro tool (https://www.ebi.ac.uk/interpro/; accessed on 20 February 2023) [83].

## Figures and Tables

**Table 1:** Putative *G. lamblia* TORC1 and TORC2 compared to their yeast and human counterparts.

Complex	*G. lamblia*	*S. cerevisiae*	*H. sapiens*
TORC1	GTOR	TOR1p or TOR2p	mTOR
	RAPTOR	Kog1p	Raptor
	LST8	Lst8p	mLST8
	-	Tco89p	-
TORC2	GTOR	TOR2p	mTOR
	-	Avo1p	mSIN1
	-	Avo2p	-
	RICTOR	Avo3p	Rictor
	LST8	Lst8p	mLST8
	-	Bit61p	-

## Data Availability

All data are available to any qualified researcher upon request from the corresponding authors.
